# Globalized parametric optimization of microwave components by means of response features and inverse metamodels

**DOI:** 10.1038/s41598-021-03095-0

**Published:** 2021-12-09

**Authors:** Anna Pietrenko-Dabrowska, Slawomir Koziel

**Affiliations:** 1 Faculty of Electronics, Telecommunications and Informatics, Gdansk University of Technology, 80-233 Gdansk, Poland; 2 Engineering Optimization and Modeling Center, Reykjavik University, 102 Reykjavik, Iceland

**Keywords:** Electrical and electronic engineering, Computational science

## Abstract

Simulation-based optimization of geometry parameters is an inherent and important stage of microwave design process. To ensure reliability, the optimization process is normally carried out using full-wave electromagnetic (EM) simulation tools, which entails significant computational overhead. This becomes a serious bottleneck especially if global search is required (e.g., design of miniaturized structures, dimension scaling over broad ranges of operating frequencies, multi-modal problems, etc.). In pursuit of mitigating the high-cost issue, this paper proposes a novel algorithmic approach to rapid EM-driven global optimization of microwave components. Our methodology incorporates a response feature technology and inverse regression metamodels to enable fast identification of the promising parameter space regions, as well as to yield a good quality initial design, which only needs to be tuned using local routines. The presented technique is illustrated using three microstrip circuits optimized under challenging scenarios, and demonstrated to exhibit global search capability while maintaining low computational cost of the optimization process of only about one hundred of EM simulations of the structure at hand on the average. The performance is shown to be superior in terms of efficacy over both local algorithms and nature-inspired global methods.

## Introduction

Topological complexity of passive microwave components has been continuously increasing over the years^1,2^. This is a consequence of growing performance demands^3^, functionality requirements^4–7^, but also miniaturization trends^8^. In the latter case, techniques such as transmission line (TL) folding^9^ or slow-wave phenomenon^10^, are often employed, leading to geometrically involved structures described by many parameters^11,12^. Circuit-theory-based methods often turn out to be inadequate in describing the intricacies of such devices. Due to electromagnetic (EM) cross-coupling and similar effects, their reliable evaluation can only be realized through full-wave EM analysis.

As a result of involved interrelations between the circuit topology and its electrical characteristics, simultaneous optimization of geometry parameters by means of numerical algorithms becomes imperative to achieve the best possible performance of the structure. In fact, numerical optimization allows proper handling of several objectives and constraints over multi-dimensional parameter spaces. Yet, it is an expensive process as even local optimization involves a considerable number of system evaluations. In many cases, including multimodal tasks^13–15^, multi-criterial design^16^, or the lack of good starting points^17,18^, globalized search is necessary, which makes the optimization problem even more challenging.

Undoubtedly, the most popular global optimization methods today are population-based nature-inspired algorithms^19–21^. Their roots can be tracked back to late 1960s^22^, and eventually dominated global search practice since 2000s^23–27^. Over the last years, the number of nature-inspired algorithms has been growing tremendously (firefly algorithm^28^, harmony search^29^, and others^30–36^). Population-based methods capitalize on exchanging information between the members of the candidate solution set^37–39^, but also generating new data using exploitative operators^40^. Avoiding local minima is facilitated by including randomness in various forms^41,42^. Typically, nature-inspired algorithms are straightforward to implement, yet, their computational efficiency is poor: a single optimization run may require from a few hundreds to many thousands of objective function evaluations, which becomes a serious problem when the system of interest is to be evaluated using full-wave EM simulation.

In high-frequency design, practical applicability of the aforementioned global search algorithms is limited to cases in which the objective function is computationally cheap (e.g., analytical array factor models for array pattern synthesis^43^), EM simulation is relatively cheap (e.g., a few seconds), or a parallelization is possible. An alternative is utilization of surrogate modelling methods^44,45^. Among many possibilities, kriging^46^, Gaussian process regression^47^, neural networks^48–50^, and polynomial chaos expansion^51^, seem to be the most popular. In a practical setup, the surrogate acts as a fast predictor, and it is often refined using the EM simulation data accumulated during the optimization process^52^. Surrogates can also be used in combination with machine learning methods^53^, or for parameter space pre-screening^54^.

The employment of data-driven surrogates in the context of global optimization is limited by the curse of dimensionality, and by nonlinearity of the microwave component responses. In practice, only devices described by a few parameters of relatively narrow ranges can be handled^55,56^. A considerable extension of the applicability range of the surrogates can be achieved using the recently proposed performance-driven modelling methods^57–59^, where the model is only constructed along a specified manifold (in the following, we use the term “manifold” to describe a curved surface in a multi-dimensional space), corresponding to designs that are optimum with respect to the assumed performance figures^57^. Constraining the domain allows for setting up reliable models over wide ranges of geometry, material, and operating parameters of the system at hand at a low computational cost. This is possible by utilizing a two-step process, where the parameter space is first mapped into a low-dimensional manifold using an auxiliary inverse model. The final surrogate is then established in the vicinity of the said manifold, which is of dramatically smaller volume than the original space. The performance-driven concept has been also generalized to variable-fidelity case^60^. Another method, recently introduced to accelerate EM-based optimization and modelling procedures, is the response feature technique^61^, where the design (modelling) objectives are expressed in terms of characteristic points of the system outputs^62^. Due to a weakly-nonlinear dependence between the characteristic point coordinates and geometry parameters, considerable savings can be achieved^61–63^.

This work discusses a novel methodology for global parameter optimization of microwave components. The proposed approach relies on identification of the most promising regions of the search space using inverse regression model set up using pre-selected random observables. The inverse model identification capitalizes on response feature technology, which is critical to handle intrinsically non-linear circuit characteristics in a computationally feasible manner. The initial design yielded by the regression model predictions is locally tuned using the trust-region gradient-based algorithm. Numerical verification of the procedure is executed using several microstrip circuits, including two rat-race couplers and a dual-band power divider. The findings confirm the global search capability of the presented framework while retaining low computational cost, comparable to strictly local optimization. At the same time, our algorithm is shown to outperform multiple-start gradient search as well as population-based metaheuristics (here, particle swarm optimization). The major novelties of the proposed globalized optimization approach include: (1) the development of a rapid procedure for identification of the promising regions of the parameter space, incorporating the response feature technology and inverse surrogates, (2) combining feature-based predictions with analytical trend functions utilized in the second stage of the search process to yield reasonable starting point for further (local) parameter tuning, (3) enabling globalized search at the cost comparable with local (e.g., gradient-based) optimization, and significantly lower than nature-inspired algorithms, (4) comprehensive demonstration of the efficacy of the method using real-world case studied, as well as benchmarking against a variety of reference methods. To the best knowledge of the authors, no similar method has been available in the literature thus far, especially in terms of combining reliability with computational efficiency. Furthermore, as the method does not rely on forward surrogate models, it is more immune to dimensionality issues than state-of-the-art (data-driven) surrogate-assisted approaches.

## Globalized microwave optimization using feature-based inverse metamodels

The purpose of this section is to introduce the optimization technique discussed in the work. Our approach employs inverse regression surrogates established using pre-selected random parameter vectors, and the characteristic points of EM simulated responses of the microwave component under design. Weakly-nonlinear relationship between these feature points and geometry parameters enables global search capability at low computational cost. The inverse model is used to render a good starting point, which is subsequently tuned by means of a local (here, gradient-based) procedure.

### Formulation of EM-driven design task

The simulation-driven design problem is formulated here as a nonlinear minimization task of the form

1 $${{\mathbf{x}}^*} = \arg \mathop {\min }\limits_{\mathbf{x}} U({\mathbf{x}},{{\mathbf{F}}_t})$$

where *U* is a scalar objective function, and ***F***_*t*_ = [*F*_*t*.1_ … *F*_*t*.*K*_]^*T*^ is a target vector of operating parameters. The objective function quantifies the quality of the design based on EM-simulated responses of the microwave component at hand, which are most often scattering parameters *S*_*kl*_(***x***,*f*), where *k* and *l* denote the corresponding ports of the circuit, ***x*** is a vector of designable parameters, and *f* is the frequency.

For the sake of example, let us consider a microwave coupler, which is to operate at the frequency *f*_0_ so that its matching and isolation characteristics, |*S*_11_| and |*S*_41_|, are minimized at *f*_0_, and the power split ratio *d*_*S*_(***x***,*f*_0_) =|*S*_21_(***x***,*f*_0_)| – |*S*_31_(***x***,*f*_0_)| reaches a target value *K*_*P*_ (e.g., 0 dB for equal power split). In this case, the operating parameter vector would be ***F***_*t*_ = [*f*_0_ *K*_*P*_]^*T*^, whereas the objective function may be defined as

2 $$ U({\mathbf{x}},{{\mathbf{F}}_t}) = U({\mathbf{x}},{[{f_0}\;{K_P}]^T}) = \max \left\{ {|{S_{11}}({\mathbf{x}},{f_0})|,|{S_{41}}({\mathbf{x}},{f_0})|} \right\} + \beta {\left[ {{d_S}({\mathbf{x}},{f_0}) - {K_P}} \right]^2} $$

where the second term is a penalty function enforcing the required power split ratio, with *β* being the penalty factor controlling the contribution of the penalty term to the overall objective function.

Another example is to design a dual-band coupler for a substrate characterized by a specific relative permittivity *ε*_*r*_, so that the circuit minimizes both |*S*_11_| and |*S*_41_| responses at the two operating frequencies *f*_0.1_ and *f*_0.2_, while providing equal power split at these frequencies. Here, the operating parameter vector is ***F***_*t*_ = [*f*_0.1_ *f*_0.2_ *ε*_*r*_]^*T*^, and the objective function may be defined as

3 $$ \begin{aligned} U({\mathbf{x}},{{\mathbf{F}}_t}) & = U({\mathbf{x}},{[{f_{0.1}}\;{f_{0.2}}\;{\varepsilon_r}]^T}) \\ & = \max \{ |{S_{11}}({\mathbf{x}},{f_{0.1}})|,|{S_{41}}({\mathbf{x}},{f_{0.1}})|,|{S_{11}}({\mathbf{x}},{f_{0.2}})|,|{S_{41}}({\mathbf{x}},{f_{0.2}})|\} \\ & \quad + \beta \left[ {{d_S}{{({\mathbf{x}},{f_{0.1}})}^2} + {d_S}{{({\mathbf{x}},{f_{0.2}})}^2}} \right] \\ \end{aligned} $$

Other design scenarios can be treated in a similar manner. This includes cases where the operating bandwidth of the device is handled explicitly (e.g., bandwidth enhancement, etc.).

### Design quality evaluation using response features

Adjustment of geometry parameters is a necessary step of microwave design process. It aims at improving the performance parameters, and, as explained in “Formulation of EM-driven design task”, it can be formulated as an optimization task, normally solved at the level of EM analysis to ensure reliability. In this work, we address globalized optimization. It is often required, either due to the lack of good starting point, or the presence of multiple local optima, some of which may fail to satisfy the prescribed performance requirements. A representative situation is a design of miniaturized structures where conventional transmission lines are replaced by CMRC or similar unit cells^10^. Therein, the relationships between geometry parameters and electrical characteristics of the cell are generally complex^12^, which renders an identification of a reasonable initial design a difficult problem. Similar issues may arise when re-designing a given circuit for operating frequencies or substrate that are away from those at the current design.

Global exploration of the parameter space is a daunting task due to nonlinearity of system characteristics (both as a function of geometry parameters and frequency), but also dimensionality issues. While direct EM-driven global search using, e.g., nature-inspired algorithms, is almost always computationally prohibitive (unless the computational model is relatively cheap to evaluate), the aforementioned reasons also hinder utilization of surrogate-assisted procedures, as rendering reliable metamodels is rarely feasible beyond a few parameters and within narrow ranges thereof.

**Figure 1:**
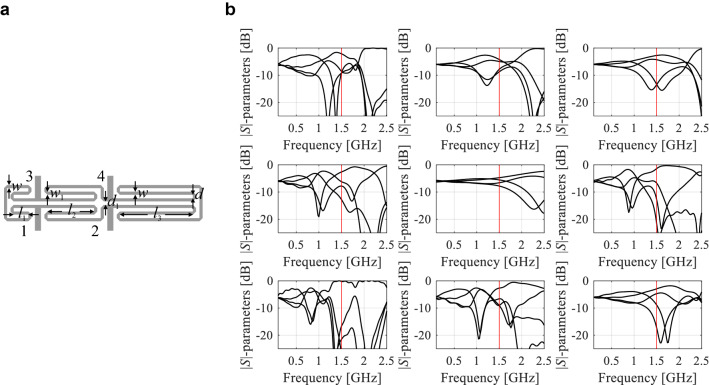
Miniaturized microstrip coupler and its scattering parameters versus frequency: (**a**) coupler geometry, (**b**) S-parameters at selected random designs within the assumed parameter space. The vertical lines mark the target operating frequency (here, 1.6 GHz). Local search carried out using the objective function such as (2) would fail when starting from most of the shown designs, due to severe misalignment between the target and the actual operating conditions.

Figure 1 illustrates several situations where a local (e.g., gradient-based) search may fail due to the lack of a good initial design or the necessity of re-designing the structure for operating conditions that are distant from those at the current design. The coupler of Fig. 1a is a compact microstrip rat-race coupler, described by six independent geometry parameters ***x*** = [*l*_1_ *l*_2_ *l*_3_ *d w w*_1_]^*T*^, further details pertaining the circuit can be found in Table 2. The example is based on one of the miniaturized coupler structures considered as verification cases in “Numerical verification”.

**Figure 2:**
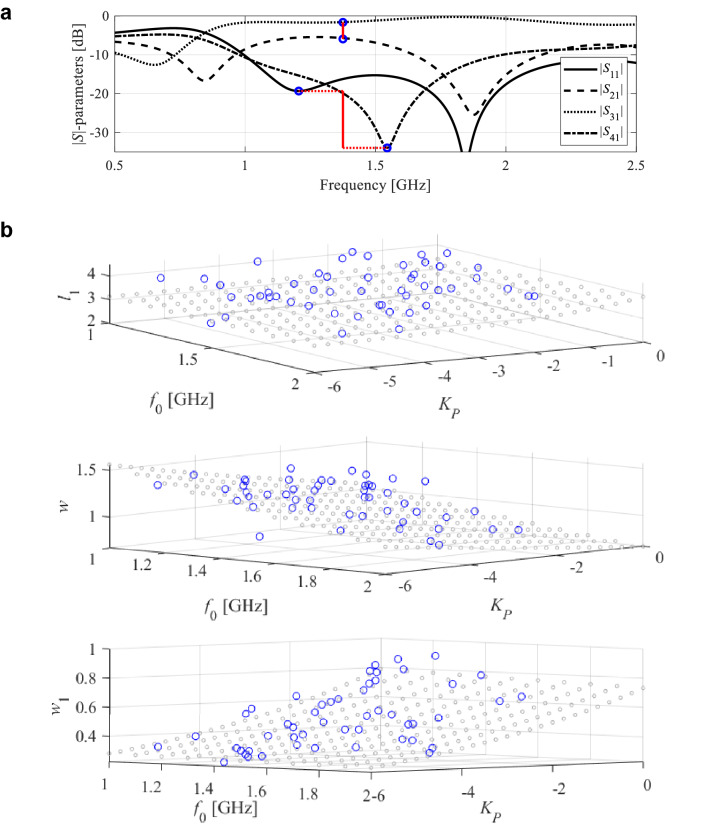
Miniaturized coupler of Fig. 1a: (**a**) response features: minima of |*S*_11_| and |*S*_41_| and power split ratio *K*_*P*_ (o); *K*_*P*_ is evaluated at the frequency (approximate operating frequency *f*_0_ of the circuit) being the average of the said minima (thick vertical line); (**b**) relationship between *f*_0_ and *K*_*P*_ and the three selected geometry parameters; the circles mark coupler designs and the gray points denote the regression model.

The issues discussed above may be mitigated by processing information extracted from the EM-simulated circuit responses in the form of the characteristic points (or response features), which is a foundation of feature-based optimization (FBO) technology^61^. FBO explores the fact that despite intrinsic nonlinearity of the system responses (cf. Fig. 1b), the relationships between the characteristic point coordinates and geometry/material parameters is much less nonlinear, which allows for obtaining a considerable amount of information about the system using a limited amount of EM simulation data, as shown in Fig. 2. Figure 2a shows the selection of the response features corresponding to minimum of matching and isolation characteristics as well as the power split ratio of miniaturized microwave coupler of Fig. 1a.

The power split *K*_*P*_ =|*S*_21_(*f*_0_)| – |*S*_31_(*f*_0_)| is evaluated at the approximate operating frequency *f*_0_ of the circuit. The frequency *f*_0_ is assessed as the average of the |*S*_11_| and |*S*_41_| minima. Note that some of the feature points may not exist depending on the particular parameter vector (e.g., the operating frequency located outside the simulation frequency range). The relationship between the operating frequency and power split ratio and the three selected geometry parameters is presented in Fig. 2b, where the specific coupler designs are shown along with the regression model of the form *a*_0_ + *a*_1_exp(*a*_2_*f*_0_ + *a*_3_*K*_*P*_), which represents the trends between the operating parameters and the circuit dimensions.

The definition of the feature points depends on the particular shape of the circuit responses and on the formulation of the design task. These could be simply the frequency and level locations of the resonances^61^, local minima/maxima of the pass-band part of the return loss^62^, or points defining a circuit bandwidth, power split, etc.^64^. In this paper, utilization of characteristic points is one of the foundations of the presented optimization technique, specifically, at its first stage (“Globalized optimization with inverse regression models”). However, the feature points are used here primarily to estimate the actual operating conditions of the circuit, rather than directly (as in FBO^61^), which was graphically illustrated in Fig. 2a.

### Globalized optimization with inverse regression models

As announced in “Design quality evaluation using response features”, this paper capitalizes on a weakly-nonlinear dependence of the geometry variables of the circuit on its operating parameters (frequency, bandwidth, power split) in order to explore the parameter space in a computationally-efficient manner. Examples of such relationships can be found in Fig. 2b for a representative miniaturized microstrip coupler. By weakly nonlinear we mean the type of relation that is usually monotonic (e.g., of the exponential type, close to proportional or inversely proportional).

This sort of relationship holds for many practical circuits, especially when the figures of interest are operating frequencies/bandwidth, or material parameters (substrate permittivity/height), but also other parameters (e.g., power split ratios for couplers). In particular, operating parameters are in typically monotonic relations with certain major parameters, despite the fact that the entire frequency characteristics may be strongly nonlinear function of frequency.

The information necessary to estimate the mentioned dependencies is acquired using randomly generated parameter vectors (observables). Some of these may be of good quality from the perspective of the assumed performance requirements, whereas others may be poor and need to be rejected. The observable quality is evaluated using the feature points extracted from the simulated scattering parameters, and comparing the utility metrics obtained this way with the design targets. The subset of the best observables is then employed to identify an inverse regression model. The latter serves two purposes: (1) to find the promising parameter space region, and (2) to generate infill points for refining the inverse surrogate. The globalized search process is executed iteratively, with a single infill point rendered per iteration, and the worsts observables replaced by those being closer to the target. The details of the procedure are explained in the remaining part of this section.

First, we introduce the notation used throughout:

- ***F***(***x***) = [*F*_1_(***x***) … *F*_*K*_(***x***)]^*T*^—a vector of the operating parameters at the design ***x*** (e.g., centre frequency, bandwidth, power split ratio), extracted from the EM simulated circuit responses. As mentioned before, the particular operating parameters are estimated based on the feature points (cf. Fig. 2a); e.g., the operating frequency of the coupler can be estimated as the average of the frequencies corresponding to the minimum of the matching and isolation characteristics. If some of the parameters cannot be extracted (e.g., some of the relevant feature points cannot be distinguished or are allocated outside the frequency range of simulation), we assign ***F***(***x***) = [0 … 0]^*T*^;
- ***L***(***x***) = [*l*_1_(***x***) … *l*_*K*_(***x***)]^*T*^—a vector of auxiliary coefficients reflecting the design quality and corresponding to the entries of the vector ***F***(***x***). For example, if the objective is to reduce the level of |*S*_11_| and |*S*_41_| at the operating frequency, the corresponding *l*_*k*_ can be the average of |*S*_11_| and |*S*_41_| at their respective minima: the lower value indicates that the design ***x*** is of higher quality. Similarly, if the operating parameter is a power split ratio, the corresponding *l*_*k*_ might be a deviation from the estimated power split and its target value. If some of the entries of ***L***(***x***) cannot be extracted, we assign ***L***(***x***) = [0 … 0]^*T*^;
- *D*(***F***,***F***_*t*_)—a function quantifying the misalignment between the target vector ***F***_*t*_ (cf. “Formulation of EM-driven design task”) and the operating parameter vector ***F***; in this work, we use *L*_2_-norm-based distance *D*(***F***,***F***_*t*_) =||***F*** – ***F***_*t*_||;
- *D*_*accept*_—user-defined control parameter utilized to terminate the global search stage of the optimization process, i.e., we assume that the current design is sufficiently close to the target if *D*(***F***,***F***_*t*_) ≤ *D*_*accept*_.

Before providing a rigorous formulation of the global search process, the following outline is discussed to clarify the operation and the meaning of the specific steps:

*Observable generation* obtain a set of parameter vectors ***x***^(*j*)^, *j* = 1, …, *N*, generated randomly over the assumed space *X* (most often, an interval defined by the lower and upper parameter bounds), typically, using a uniform probability distribution. The vectors are generated as long as necessary to obtain *N* designs for which ||***F***(***x***^(*j*)^)||> 0, *j* = 1, …, *N*.
2. *Inverse model construction* Using the set of triples {***F***(***x***^(*j*)^), ***L***(***x***^(*j*)^), ***x***^(*j*)^}_*j* = 1,…,*N*_, identify an inverse regression model *r*_*I*_(***F***) with the values in *X*; the model quantifies the dependence between the operating and geometry parameters of the circuit. The analytical formulation of *r*_*I*_ will be discussed later in the section;
*Design prediction* Employ the inverse model *r*_*I*_ to identify a candidate parameter vector ***x***_*tmp*_ = *r*_*I*_(***F***_*t*_), where ***F***_*t*_ is the vector of target operating parameters (cf. “Formulation of EM-driven design task”). If ||***F***(***x***_*tmp*_)||> 0 and *D*(***F***(***x***_*tmp*_),***F***_*t*_) < max{*j* = 1, …, *N* : *D*(***F***(***x***^(*j*)^),***F***_*t*_)}, replace the vector realizing the above maximum by ***x***_*tmp*_, and reset *r*_*I*_.

The second and the third step are iterated in the attempt to find a design that is sufficiently close to the target. More specifically, the procedure is terminated if *D*(***F***(***x***_*tmp*_),***F***_*t*_) < *D*_max_ (a user-defined acceptance threshold). The next stage is local optimization as described in “Local optimization procedure”. It can be noted that the procedure generates random parameter vectors until a sufficient number of designs are found for which clearly defined feature points can be extracted. This is followed by constructing the inverse model (see Fig. 2b for a graphical illustration), which is then used as a predictor to yield a location of the design for which the operating parameters are possibly close to the target ***F***_*t*_. If the candidate design is of sufficient quality (according to function *D*(***F***,***F***_*t*_)), it replaces the worst of the existing base vectors.

**Figure 3:**
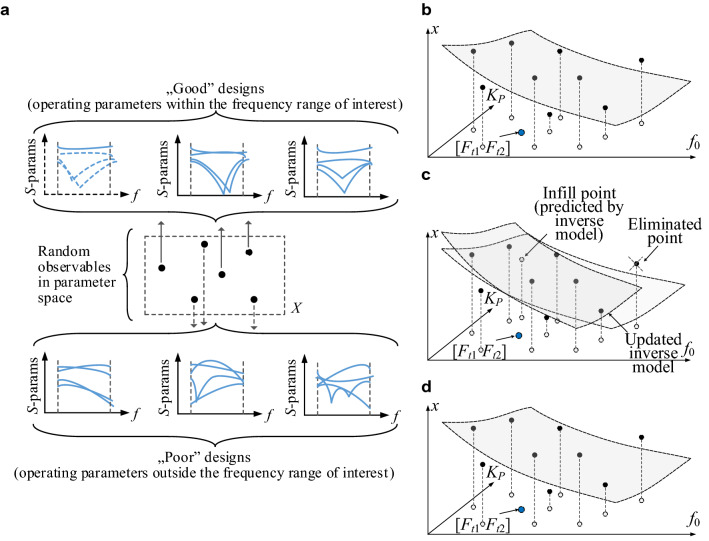
Fundamental components of the proposed optimization procedure: (**a**) selection of the “good” observables, (**b**) observables (•) in the two-dimensional parameter space *f*_0_, *K*_*P*_ for a selected geometry parameter ***x***; the observable projections onto the *f*_0_-*K*_*P*_ plane, the initial inverse model (grey surface); the target operating parameters (blue circle); (**c**) first iteration: the infill point predicted by *r*_*I*_ (grey circle) replaces the worst observable and *r*_*I*_ is updated; (**d**) last iteration: the observables concentrated near the target operating parameters and the inverse model yields the design sufficiently close to the target.

Because the design replacement in the dataset {***x***^(*j*)^} is governed by the proximity function *D*(***F***,***F***_*t*_), over time, the inverse model will be focused on the region containing designs that exhibit low values thereof. For the same reason, the local accuracy of the surrogate will gradually improve. A graphical illustration of the procedure can be found in Fig. 3. The acquisition of the observables is carried out as follows: only the designs with their corresponding operating parameters (centre frequency, power split ratio) within the region of interest and the simulation frequency range will contribute to a construction of the inverse model *r*_*I*_ (see Fig. 3a).

The inverse regression model *r*_*I*_(***F***) is the fundamental component of the proposed optimization procedure. As outlined above, it is constructed using the triples {***F***(***x***^(*j*)^), ***L***(***x***^(*j*)^), ***x***^(*j*)^}_*j* = 1,…,*N*_. The analytical form of *r*_*I*_ can be simple because the dependence between the geometry parameters and the operating conditions of the circuit is typically only weakly nonlinear. Nevertheless, the model has to have a sufficient flexibility to account for the fact that the aforementioned dependence may be close to inverse proportionality for certain parameters. Having this in mind, the following form has been assumed:

4 $$ {{\mathbf{r}}_I}({\mathbf{F}}) = {{\mathbf{r}}_I}\left( {\left[ {\begin{array}{*{20}{c}} {f_1} \\ {...} \\ {f_K} \end{array}} \right]} \right) = \left[ \begin{gathered} {r_{I.1}}({\mathbf{F}}) \\ \cdots \\ {r_{I.n}}({\mathbf{F}}) \\ \end{gathered} \right] = \left[ \begin{gathered} {p_{1.0}} + {p_{1.1}}{e^{\sum\nolimits_{k = 1}^K {{p_{1.k + 1}}{f_k}} }} \\ \cdots \\ {p_{n.0}} + {p_{n.1}}\,{e^{\sum\nolimits_{k = 1}^K {{p_{n.k + 1}}{f_k}} }} \\ \end{gathered} \right]$$

The surrogate is identified by solving

5 $$ \left[ {{p_{j.0}}\;{p_{j.1}}\;...\;{p_{j.K + 1}}} \right] = \arg \mathop {\min }\limits_{[{b_0}\;{b_1}\;...\;{b_{K + 1}}]} \sum\limits_{k = 1}^N {{w_k}{{\left[ {{r_{I.j}}\left( {{\mathbf{F}}({{\mathbf{x}}^{(k)}})} \right) - x_j^{(k)}} \right]}^2}} ,j = \, 1, \, \ldots ,n $$

where ***x***^(*j*)^ = [*x*_1_^(*j*)^ … *x*_*n*_^(*j*)^]^*T*^. The weighting factors *w*_*k*_ are computed based on the auxiliary vectors ***L***(***x***^(*j*)^)

6 $$ {w_k} = {\left[ {1 - \max \{ {l_1}({{\mathbf{x}}^{(j)}}),...,{l_k}({{\mathbf{x}}^{(j)}})\} } \right]^2},\;\;\;k = \, 1, \, \ldots ,N $$

Here, it is assumed that the components *l*_*k*_ are normalized, and can only assume the values from the interval [0, 1], with zero corresponding to the highest-quality design (with respect to the *k*th operating parameter), and one corresponding to the lowest-quality design. In the example considered before, with the objective being to reduce the level of |*S*_11_| and |*S*_41_| at the operating frequency, the corresponding *l*_*k*_ could be selected as the average of |*S*_11_| and |*S*_41_| at their respective minima. Clearly, good design corresponds to *l*_*k*_ close to zero (low reflection and high isolation), whereas poor design would be associated with *l*_*k*_ closer to one.

Overall, the inverse regression model *r*_*I*_ is essentially a trend function that approximates the observable set {***x***^(*j*)^} in the weighted *L*-square sense (cf. (2)). The reason for introducing the weights *w*_*k*_ is to discriminate between the low- and high-quality observables so that the latter affect *r*_*I*_ to a greater extent.

It should also be noted that, in general, construction of the inverse models may be hindered by non-uniqueness issues (e.g., Refs.^71,72^). Notwithstanding, for typical microwave passive components, the operating conditions (e.g., operating frequency, bandwidth, etc.) are mainly dependent on specific geometry parameters controlling electrical lengths of its parts, therefore, the relationship between designable parameters and operating conditions is usually monotonic. Although it might not be so for compact structures (especially those utilizing slow-wave phenomenon), the mentioned major parameters enforce monotonicity. Furthermore, the inverse model is established as a regression surrogate, so it does not follow exactly its training data but only the trend. Consequently, it accommodates possible non-uniqueness due to the parameters of minor importance (from the point of view of the trends). Finally, it should be noted that identification of the inverse model is a weighted regression task (cf. (5)), with less weight put on “poor” observables, thereby, extracting the trends from the best points only.

Figure 3b provides a graphical illustration of the inverse model for the microstrip coupler of Fig. 1a. Whereas the conceptual illustration of the global search process is presented in Fig. 3c,d. In the first iteration of this search (cf. Fig. 3c), the infill point predicted by *r*_*I*_ replaces the worst observable and the model *r*_*I*_ is updated. Figure 3d presents the last iteration, in which the observables are concentrated near the target operating parameters, hence the updated inverse model is capable of yielding the design which is sufficiently close to the target. Hence, the procedure may be terminated and followed by a local optimization (“Local optimization procedure”). Observe that Fig. 3b–d refer to a single geometry parameter *x*; the same scheme is applied to all parameters simultaneously.

**Figure 4:**
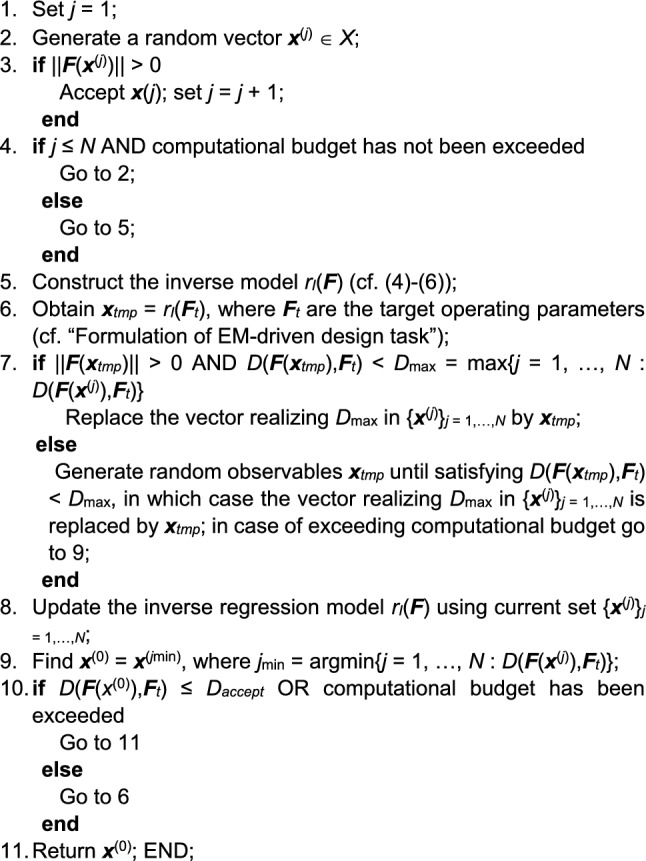
Pseudocode of the globalized optimization of microwave components using inverse regression model. The presented flow represents the first (global) optimization stage, which is followed by a local optimization (cf. “Local optimization procedure”).

The operating flow of the global search process as proposed in this work has been summarized in Fig. 4 in the form of a pseudocode. Therein, Steps 1 through 4 correspond to identification of *N* observables with their operating parameters being within the prescribed ranges, in particular, the frequency-related parameters being within the range of circuit simulation. These parameter sets are employed in Step 5 to construct the inverse model *r*_*I*_. The remaining steps describe utilization of *r*_*I*_ for generating a candidate design ***x***_*tmp*_, its evaluation, and insertion into the observable pool (provided it is of sufficient quality). These are followed by rebuilding the inverse model.

The termination condition is *D*(***F***(***x***^(0)^),***F***_*t*_) ≤ *D*_*accept*_, i.e., identification of a design, which is sufficiently close to the target ***F***_*t*_, which then becomes a starting point ***x***^(0)^ for local optimization (cf. “Local optimization procedure”). If such a design cannot be found, the procedure is terminated upon exceeding its computational budget, which results in returning the best design found so far.

### Local optimization procedure

Global search procedure, as described in “Globalized optimization with inverse regression models”, yields a design ***x***^(0)^ satisfying the condition *D*(***F***(***x***^(0)^),***F***_*t*_) ≤ *D*_*accept*_, with the threshold *D*_*accept*_ assigned to make sure that the operating parameters at ***x***^(0)^ are sufficiently close to ***F***_*t*_ to make the target attainable by means of local optimization. In this work, it is realized using the trust-region (TR) gradient-based algorithm with numerical derivatives^65^. The TR algorithm produces a series of approximations to the optimum design x^*^, denoted as ***x***^(*i*)^, *i* = 0, 1, … The subsequent iteration points are obtained as

7 $$ {{\mathbf{x}}^{(i + 1)}} = \arg \mathop {\min }\limits_{{\mathbf{x}};\; - {{\mathbf{d}}^{(i)}} \leqslant {\mathbf{x}} - {{\mathbf{x}}^{(i)}} \leqslant {{\mathbf{d}}^{(i)}}} {U_L}({\mathbf{x}},{{\mathbf{F}}_t})$$

where the objective function *U*_*L*_ takes the same form as the function *U* (cf. (1)); however, it is computed using of the first-order Taylor expansion model ***G***^(*i*)^(***x***,*f*) of the system responses established at the current point ***x***^(*i*)^. The linear model is defined, for the *S*-parameter *S*_*kl*_, as

8 $$ {G^{\left( i \right)}}\left( {x,f} \right) \, = {S_{kl}}\left( {{x^{(i)}},f} \right) \, + {\nabla_{Skl}}\left( {{x^{(i)}},f} \right) \times \left( {x--{x^{(i)}}} \right)$$

The gradients in (8) are estimated using finite differentiation. The trust region in (7) is an interval [***x***^(*i*)^ – ***d***^(*i*)^, ***x***^(*i*)^ + ***d***^(*i*)^]. The size vector ***d***^(*i*)^ is adjusted using the standard TR rules^65^. The candidate vector ***x***^(*i*+1)^ is accepted if it reduces the objective function value at the EM simulation level, i.e., if *U*(***x***^(*i*+1)^,***F***_*t*_) < *U*(***x***^(*i*)^,***F***_*t*_). Otherwise, it is rejected and the iteration is repeated with reduced ***d***^(*i*)^.

The algorithm termination is determined by the convergence in argument ||***x***^(*i*+1)^ – ***x***^(*i*)^||< ε, or diminishing the TR size, i.e., ||***d***^(i)^||< *ε* (whichever occurs first). Here, we use *ε* = 10^–3^. In order to reduce the computational cost of the optimization process, finite differentiation (normally entailing n additional EM analysis of the circuit per iteration) is replaced by the rank-one Broyden formula^66^ when close to convergence, specifically, if ||***x***^(*i*+1)^ – ***x***^(*i*)^||< 10*ε*.

### Global optimization framework

**Figure 5:**
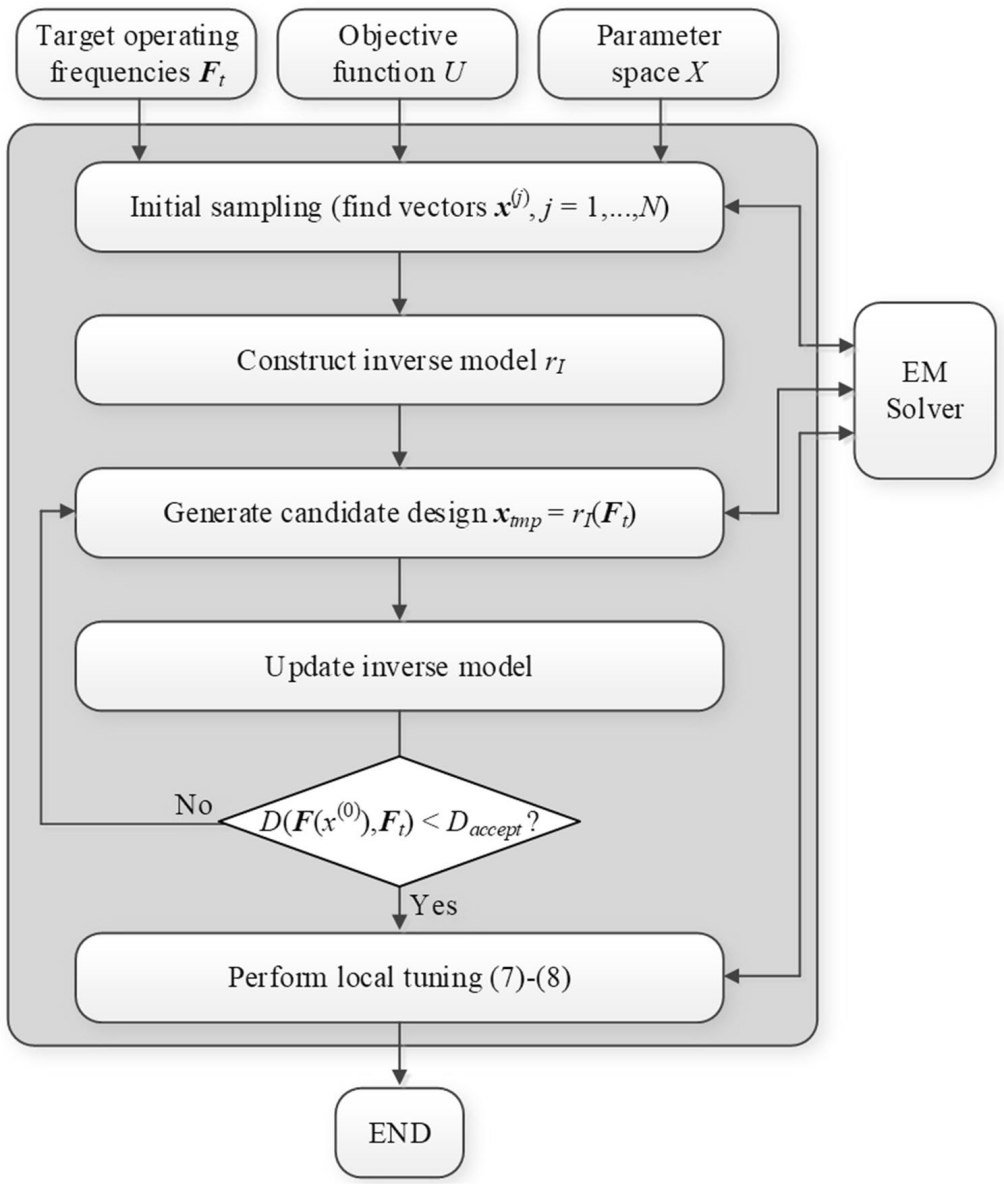
Flow diagram of the proposed framework for globalized optimization of microwave components.

The operating flow of the globalized optimization framework discussed in this work is summarized below (see also Fig. 5). Its two main components are the global and local optimization procedures formulated in “Globalized optimization with inverse regression models” and “Local optimization procedure”, respectively. The framework uses the control parameters gathered in Table 1. Note that only the first two parameters (primary parameters of Table 1), i.e., *N* and *D*_*accept*_, are specific to the proposed technique, whereas the remaining ones are conventional (in terms of numerical optimization routines). Parameter *N* (i.e., the number of observables utilized for inverse model setup) should be of the same order as design space dimensionality but also take into account the number of design objectives in order to properly account for the geometry of the set comprising high-quality designs. An appropriate value of *D*_*accept*_ is problem-specific and should be set to make the target operating parameters attainable by means of a local algorithm (at the local optimization stage). In practice, operating parameters most often relate to the operating frequency (or frequencies) of a component under design. In such a case, it suffices to set *D*_*accept*_ equal to approximately half of the intended operating bandwidth, which, in turn, typically ranges from a dozen to few dozen percent of the operating frequency. Whereas the parameters *N*_max.*k*_, *k* = 1, 2, 3, should be set up with some margin in order to ensure the algorithm termination due to convergence rather than exceeding the budget. Thus, *N*_max.1_ and *N*_max.2_ should be of one order of magnitude larger than the design space dimensionality, and *N*_max.3_ a few times higher.

**Table 1:** Control parameters of the proposed framework for global microwave design optimization along with their suggested values.

Parameter rating	Parameter symbol	Explanation	Default value
Primary	*N*	Number of observables for inverse model construction	10
	*D*_*accept*_	Threshold for accepting designs produced by the global search stage (cf. “Globalized optimization with inverse regression models”)	0.2
Secondary	*N*_max.1_	Computational budget: maximum number of EM evaluations for initial sampling	100
	*N*_max.2_	Computational budget: maximum number of EM evaluations for global search stage	100
	*N*_max.3_	Computational budget: maximum number of EM evaluations for local optimization stage	500
	ε	Termination threshold (for convergence in argument and trust-region size, cf. “Local optimization procedure”)	0.001

In short, the operating flow of the entire optimization process can be described using the following three stages:

1. *Input argument setup*:
  Target operating frequencies ***f***_*t*_,
  Objective function *U*,
  Parameter space *X*;
2. *Global search*: Obtain initial design ***x***^(0)^ by performing the algorithm of “Globalized optimization with inverse regression models”;
3. *Local optimization*: Find the final design x^*^ using the TR algorithm of “Local optimization procedure”.

The flow diagram of the process can be found in Fig. 5, where the first stage is broken down into several components, whereas the local refinement is represented by a single block. It should be emphasized that in this work, the global search procedure does not involve any direct global optimization algorithm. Instead, identification of the approximate allocation of the globally optimum solution is obtained from the inverse model. Its evaluation at the target objectives directly brings us to the appropriate portions of the parameter space. The key factor behind the efficacy of this approach is that the objective space is normally of considerably lower dimension than the parameter space, which allows us to construct a relatively accurate inverse model using a limited number of observables. These operating principles are the major differences between the methodology proposed in this work and the majority of approaches to global optimization of expensive simulation models reported in the literature.

### Numerical verification

Here, the globalized optimization framework introduced in “Globalized microwave optimization using feature-based inverse metamodels” is validated and its performance is demonstrated using several examples of microstrip components. These include two miniaturized couplers and a dual-band power divider. The verification results are supplemented by comparisons with multiple-start local optimization (to validate the need for global search) and a state-of-the-art population-based metaheuristic algorithm (to corroborate the efficacy of the presented approach).

The geometries of the test structures are introduced in “Case studies” along with the formulations of the respective design problems. “Setup and results” showcases the numerical results obtained using the proposed and the benchmark methods. “Discussion” provides a summary and discussions thereof.

### Case studies

**Figure 6:**
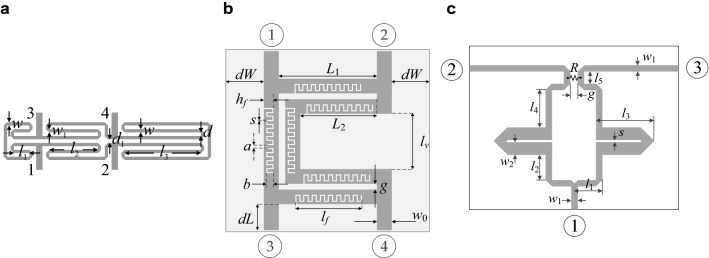
Microstrip components used for verification experiments: (**a**) rat-race coupler with folder transmission lines (RRC1)^67^, (**b**) rat-race coupler coupler with defected microstrip structure (RRC2)^68^, (**c**) dual-band power divider (PD), lumped resistor denoted as R^69^.

Numerical verification of the presented optimization procedure has been carried out using three microstrip circuits shown in Fig. 6. We have:

- Circuit I: a compact microstrip rat-race coupler (RRC1)^67^, implemented on RO4003 substrate (*ε*_*r*_ = 3.38, *h* = 0.762 mm). The adjustable geometry parameters are ***x*** = [*l*_1_ *l*_2_ *l*_3_ *d w w*_1_]^*T*^; the remaining parameters are *d*_1_ = *d* +|*w* – *w*_1_|, *d* = 1.0, *w*_0_ = 1.7, and *l*_0_ = 15 fixed (dimensions in mm). For this circuit, the goal is to minimize the matching and isolation characteristics, |*S*_11_| and |*S*_41_|, at the intended operating frequency *f*_0_, while ensuring a required power split ratio *K*_*P*_. The objective function is defined as in (2) (“Formulation of EM-driven design task”).
- Circuit II: a compact rat-race coupler (RRC2) using a defected microstrip structure (meander spurline) within a folded transmission line, implemented on 0.15-mm-thick substrate^68^. The adjustable parameters are ***x*** = [*L*_1_ *b*_*r*_* g h*_*fr*_* s l*_*fr*_]^*T*^. The dimensions are in mm except for the relative quantities denoted using the subscript *r*; these parameters are unitless. The following relationships hold: *L*_2_ = *L*_1_ – *g* – *w*_0_*, a* = (*l*_*f*_* –* 17* s*)/16, *b* = (*h*_*f*_ – *s*)*b*_*r*_, *l*_*f*_ = *L*_2_ *l*_*fr*_, *l*_*v*_ = *L*_1_ – 2* g* – *2w*_0_, and *h*_*f*_ = *s* + (*w*_0_ – *s*)*h*_*fr*_; *dW* = *dL* = 10 mm. The input line width *w*_0_ is computed for a given substrate permittivity *ε*_*r*_ so as to ensure 50 Ω input impedance. The design goal is—for a given the substrate permittivity *ε*_*r*_—to minimize the matching and isolation characteristics, |*S*_11_| and |*S*_41_|, at the intended operating frequency *f*_0_, while ensuring equal power split. The objective function is defined as in (2) but with *K*_*P*_ = 0 dB.
- Circuit III: a dual-band equal-split power divider (PD)^69^, implemented on AD250 substrate (*ε*_*r*_ = 2.5, *h* = 0.81 mm). The adjustable geometry parameters are ***x*** = [*l*_1_ *l*_2_ *l*_3_ *l*_4_ *l*_5_ *s w*_2_]^*T*^ (dimensions in mm); *w*_1_ = 2.2 mm and *g* = 1 mm are fixed. The design goal is to simultaneously minimize the input matching |*S*_11_|, output matching |*S*_22_|, |*S*_33_|, as well as isolation |*S*_23_| simultaneously at two operating frequencies *f*_1_ and *f*_2_. The objective function is similar to (3) but the equal power split condition is not directly handled in the optimization process as it is implied by the structure symmetry.

The computational models for all three circuits are implemented in CST Microwave Studio, and simulated using the time-domain solver. Table 2 provides information about the specific design tasks considered (i.e., the target values of the operating parameters), as well as the lower and upper bounds for parameters. It should be noted that parameter ranges are very wide with the average ratio of the upper-to-lower bound being 11.7, 4.6, and 10.3 for Circuit I, II, and III, respectively. The reason for selecting such broad ranges of geometry parameters was to ensure that the considered design tasks are sufficiently challenging, as well as to emulate the scenario under which the designer does not have a clear indication of what a good initial design should be, thereby to shift most of the design decisions concerning the starting point allocation to the optimization algorithm itself, rather than to engage the expert knowledge.

**Table 2:** Target operating parameters and parameter spaces for circuits I through III.

Circuit	Target operating parameters		Parameter space *X* (lower bounds *l *and upper bounds *u*)
	Symbols	Specific values for numerical experiments	
I	***F***_*t*_ = [*f*_0_ *K*_*P*_]^*T*^	Case 1: *f*_0_ = 1.8 GHz, *K*_*P*_ = –3 dB	***l*** = [0.5 5.0 5.0 0.2 0.2 0.2]^*T*^ ***u*** = [15.0 30.0 50.0 2.0 2.0 2.0]^*T*^
		Case 2: *f*_0_ = 1.2 GHz, *K*_*P*_ = 0 dB	
II^a^	***F***_*t*_ = [*f*_0_]^*T*^	Case 1: *f*_0_ = 1.5 GHz, *ε*_*r*_ = 2.5	***l*** = [20.0 0.1 1.0 0.2 0.2 0.2]^*T*^ ***u*** = [40.0 0.95 5.0 0.95 0.5 0.8]^*T*^
		Case 2: *f*_0_ = 1.2 GHz, *ε*_*r*_ = 4.4	
III	***F***_*t*_ = [*f*_1_ *f*_2_]^*T*^	Case 1: *f*_1_ = 3.0 GHz, *f*_2_ = 4.8 GHz	***l*** = [10.0 1.0 10.0 0.5 1.0 0.1 1.5]^*T*^ ***u*** = [40.0 20.0 40.0 15.0 6.0 1.5 8.0]^*T*^
		Case 2: *f*_1_ = 2.0 GHz, *f*_2_ = 3.3 GHz	

^a^The circuit is to be optimized for a specific substrate of given relative permittivity *ε*_*r*_*.*

### Setup and results

Table 3 shows the experimental setup for the optimization framework proposed in this work and the benchmark methods. These include particle swarm optimization, employed as a representative population-based metaheuristic, as well as the trust-region gradient-based algorithm (as described in “Local optimization procedure”) with random initial designs. The TR procedure is considered to demonstrate that local search is insufficient for the considered design task and often fails when the initial design is away from the target.

**Table 3:** Experimental setup: Proposed optimization framework and the benchmark.

Method	Control parameters	Termination condition	Comments
Inverse-model-based algorithm (this work)	*N* = 10, *N*_max.1_ = 100, *N*_max.2_ = 100, *N*_max.3_ = 500, *D*_*accept*_ = 0.2	*ε* = 10^−3^	cf. “Globalized microwave optimization using feature-based inverse metamodels”
PSO^70^	Population size 10 *χ* = 0.73, *c*_1_ = *c*_2_ = 2.05	Maximum number of iterations (100)	Computational budget limited to 1000 EM simulations due to high computational cost of numerical experiments
TR gradient search	Standard setup (e.g.,^65^)	*ε* = 10^−3^	Gradients estimated using finite differentiation; Termination based on convergence in arguments OR reducing the TR size

**Figure 7:**
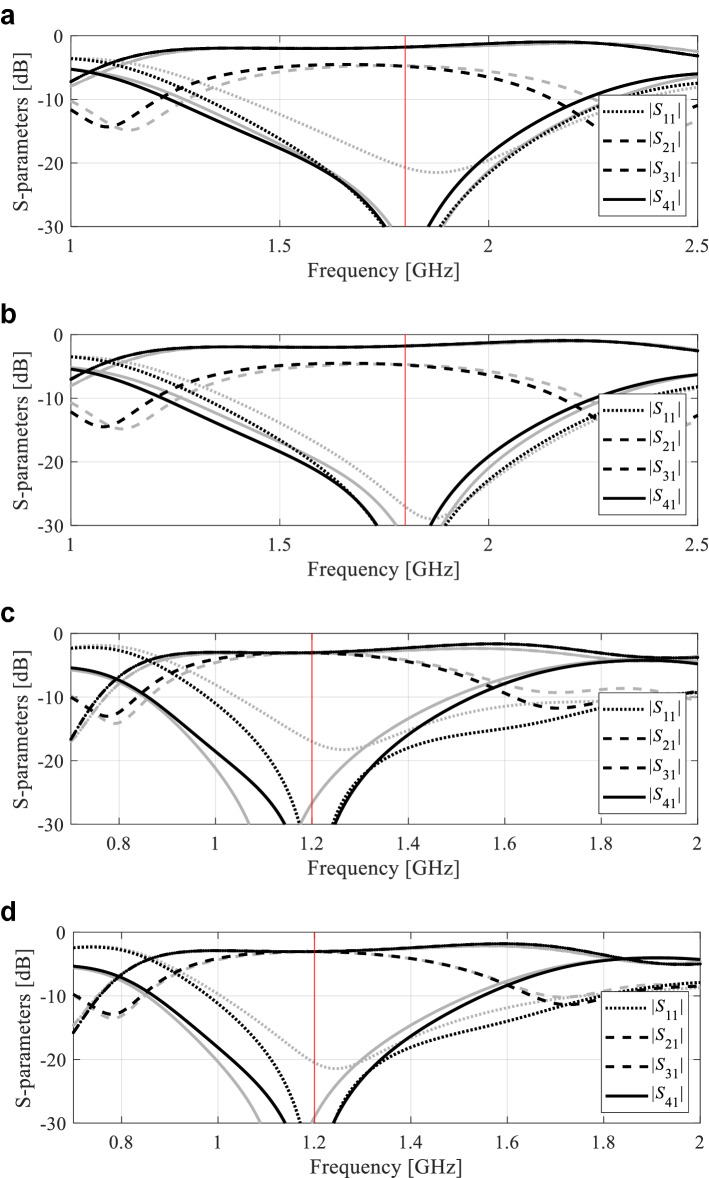
Circuit I: S-parameter characteristics at the optimized designs found by the proposed global optimization framework for two selected algorithm runs, Case 1: (**a**,**b**) Designs 1 and 2, respectively, Case 2: (**c**,**d**) Designs 1 and 2, respectively. Gray lines correspond to the initial design ***x***^(0)^ obtained using the global search stage, black lines represent the responses at the final design. Vertical lines mark the target operating frequencies.

**Figure 8:**
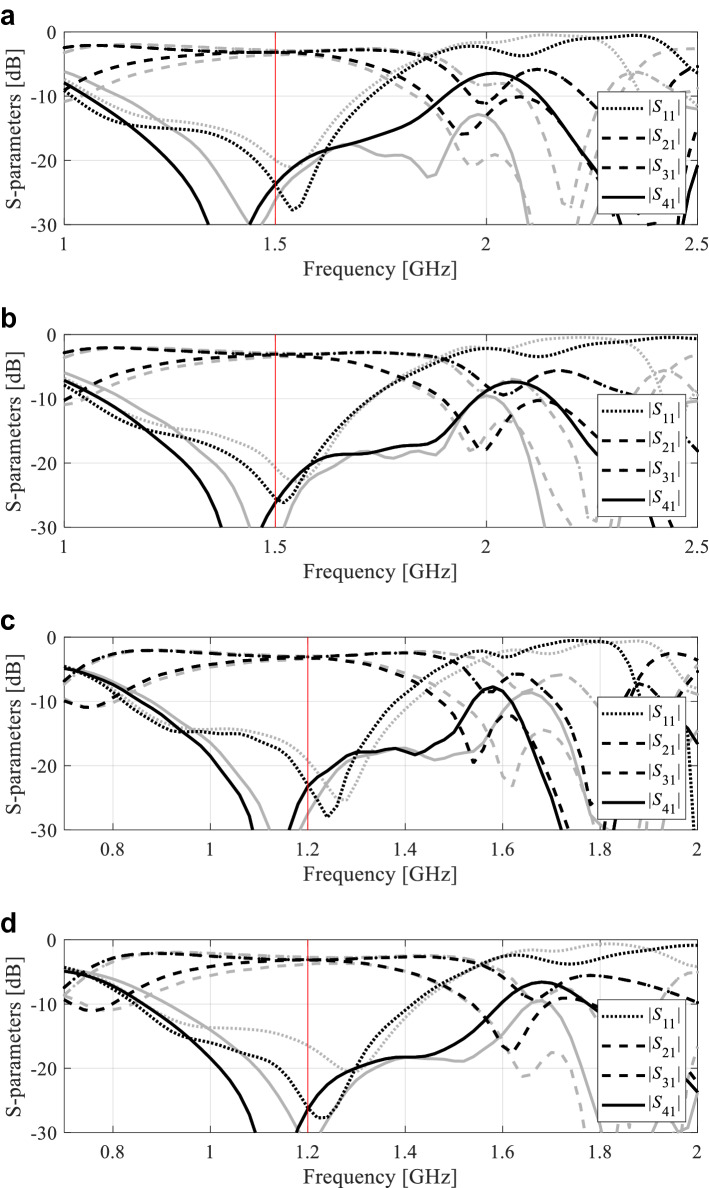
Circuit II: S-parameter characteristics at the optimized designs found by the proposed global optimization framework for two selected algorithm runs, Case 1: (**a**,**b**) Designs 1 and 2, respectively, Case 2: (**c**,**d**) Designs 1 and 2, respectively. Gray lines correspond to the initial design ***x***^(0)^ obtained using the global search stage, black lines represent the responses at the final design. Vertical lines mark the target operating frequencies.

**Figure 9:**
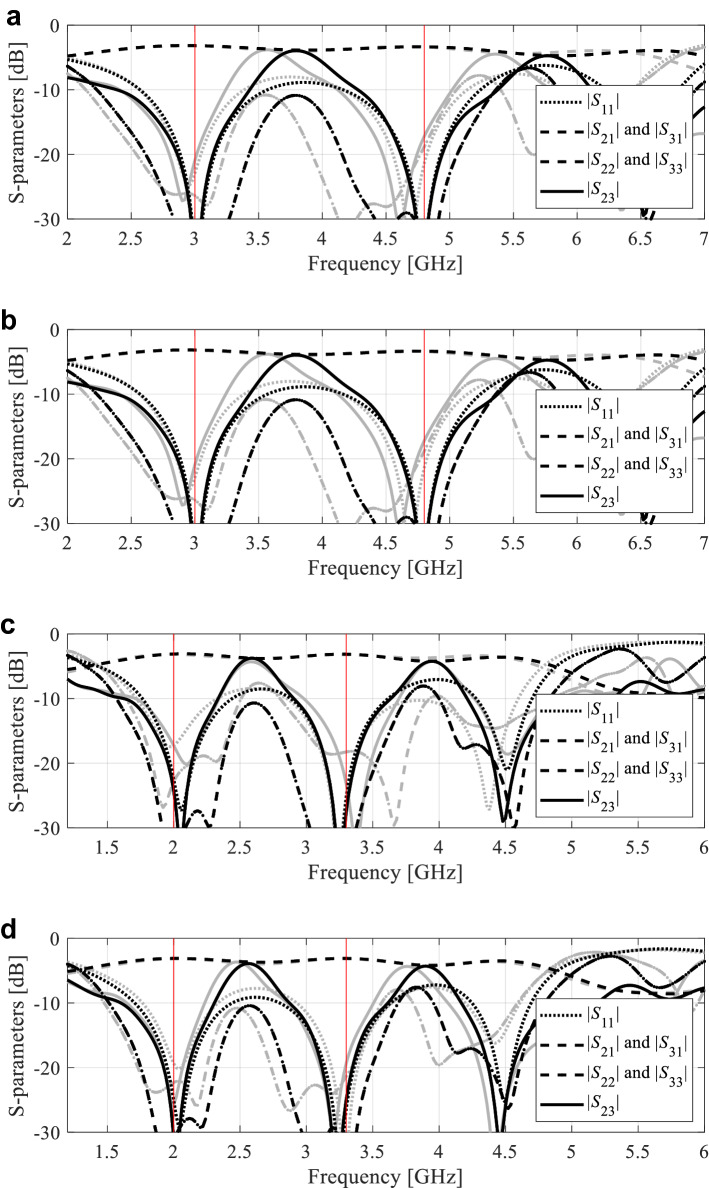
Circuit III: S-parameter characteristics at the optimized designs found by the proposed global optimization framework for two selected algorithm runs Case 1: (**a**,**b**) Designs 1 and 2, respectively, Case 2: (**c**,**d**) Designs 1 and 2, respectively. Gray lines correspond to the initial design ***x***^(0)^ obtained using the global search stage, black lines represent the responses at the final design. Vertical lines mark the target operating frequencies.

The numerical results are provided in Tables 4, 5 and 6 for Circuit I, II, and III respectively. The scattering parameter responses at the designs obtained using the proposed framework for selected algorithm runs can be found in Figs. 7, 8 and 9.

**Table 4:** Circuit I: optimization results.

Verification case	Optimization method	Inverse-surrogate-based algorithm (this work)	PSO		TR gradient-based algorithm
			50 iterations	100 iterations	
Case 1: *f*_0_ = 1.8 GHz, *K*_*P*_ = − 3 dB	Average objective function value [dB]	− 36.7	− 24.8	− 34.0	− 18.7
	Computational cost^a^	125.8	500	1000	102.8
	Success rate^b^	10/10	9/10	10/10	6/10
Case 2: *f*_0_ = 1.2 GHz, *K*_*P*_ = 0 dB	Average objective function value [dB]	− 39.9	− 23.7	− 36.2	48.3
	Computational cost^a^	130.5	500	1000	68.7
	Success rate^b^	10/10	9/10	10/10	5/10

^a^The cost expressed in terms of the number of EM simulations of the antenna structure under design.

^b^Number of algorithms runs at which the operating parameters were allocated to satisfy the condition *D*(***F***(x*),***f***_*t*_) ≤ *D*_*accept*_*.*

**Table 5:** Circuit II: optimization results.

Verification case	Optimization method	Inverse-surrogate-based algorithm (this work)	PSO		TR gradient-based algorithm
			50 iterations	100 iterations	
Case 1: *f*_0_ = 1.5 GHz, *ε*_*r*_ = 2.5	Average objective function value [dB]	− 18.6	− 17.6	− 19.2	− 1.8
	Computational cost^a^	85.5	500	1000	77.0
	Success rate^b^	10/10	10/10	10/10	5/10
Case 2: *f*_0_ = 1.2 GHz, *ε*_*r*_ = 4.4	Average objective function value [dB]	− 21.5	− 19.4	− 22.5	7.6
	Computational cost^a^	90.2	500	1000	83.8
	Success rate^b^	10/10	9/10	10/10	5/10

^a^The cost expressed in terms of the number of EM simulations of the antenna structure under design.

^b^Number of algorithms runs at which the operating parameters were allocated to satisfy the condition *D*(***F***(x*),***f***_*t*_) ≤ *D*_*accept*_*.*

**Table 6:** Circuit III: optimization results.

Verification case	Optimization method	Inverse-surrogate-based algorithm (this work)	PSO		TR gradient-based algorithm
			50 iterations	100 iterations	
Case 1: *f*_0_ = 1.5 GHz, *ε*_*r*_ = 2.5	Average objective function value [dB]	− 33.9	− 19.6	− 18.8	− 12.3
	Computational cost^a^	99.1	500	1000	95.1
	Success rate^b^	10/10	8/10	9/10	2/10
Case 2: *f*_0_ = 1.2 GHz, *ε*_*r*_ = 4.4	Average objective function value [dB]	− 23.6	− 18.8	− 19.7	− 20.6
	Computational cost^a^	99.2	500	1000	93.8
	Success rate^b^	10/10	8/10	9/10	7/10

^a^The cost expressed in terms of the number of EM simulations of the antenna structure under design.

^b^Number of algorithms runs at which the operating parameters were allocated to satisfy the condition *D*(***F***(x*),***f***_*t*_) ≤ *D*_*accept*_*.*

The breakdown of the computational cost of the proposed algorithm is the following (averaging over ten algorithm runs):

- Circuit I: average cost of global search stage was 40 EM simulations; average number of TR iterations was 10, with the average cost of each iteration of about 8.8 EM simulations (each iteration involved six extra analyses for Jacobian estimation);
- Circuit II: average cost of global search stage was 22 EM simulations; average number of TR iterations was 8, with the average cost of each iteration of about eight EM simulations;
- Circuit III: average cost of global search stage was 31 EM simulations; average number of TR iterations was seven with the average cost of each iteration of about ten EM simulations.

For the TR gradient-based algorithm (last column of Tables 4, 5 and 6), the cost breakdown is the following:

- Circuit I: average number of TR iterations was eleven, with the average cost of each iteration of about eight EM simulations;
- Circuit II: average number of TR iterations was nine, with the average cost of each iteration of about nine EM simulations;
- Circuit III: average number of TR iterations was 11, with the average cost of each iteration of about nine EM simulations.

## Discussion

The results gathered in “Setup and results” allow us to draw several conclusions concerning the presented optimization strategy, not only in terms of its efficacy and the computational complexity but also how its performance compares to the benchmark methods. These are the main points:

- The presented approach does exhibit a global search capability, which is corroborated by the fact that satisfactory designs have been found in all runs of the algorithm (ten per case). Also, as illustrated in Figs. 7, 8 and 9, the parameter vectors x(0) found by the global search stage are of good quality (in particular, with the operating frequency being close to the target), which makes local optimization sufficient. On the contrary, the benchmark local search from random initial designs often fails (at about fifty percent of the cases), and its performance is highly dependent on the initial design quality. Consequently, the average value of the optimized objective function is considerably worse than for the proposed method. Population-based metaheuristics (here, PSO) performs much better, but its computational complexity is high. It can also be observed that there is a noticeable difference in the design quality produced after 50 and 100 PSO iterations. This indicates that computational budget set at 500 EM simulations is insufficient for this method.
- The quality of the designs obtained using the presented technique surpasses that obtained using both local search and PSO. Although local optimization may produce a design that is of similar quality, it only happens when the initial points was sufficiently close to the target. Clearly, for the algorithm presented in this work, the problem of initial design has been eliminated, which makes it considerably more robust.
- In terms of computational efficiency, the presented approach compares favorably with the population-based algorithm as the average running cost is only about 128, 88, and 99 EM analyses of the system for Circuit I, II, and III, respectively. At the same time, our methodology is only slightly more expensive than local optimization (by 23 percent on the average across all three circuits considered).
- This is because the cost of the first (global) search stage is low, only 40, 22, and 31 EM simulations on the average for Circuit I, II, and III, respectively. This level of efficiency is a result of combining the response feature technology with inverse modeling, in particular, establishing the model over low-dimensional operating condition space. The latter requires a limited number of samples to identify relationships between the operating frequency, power split, etc., in a reliable manner.

As demonstrated, the presented technique offers both global search capability and computational efficiency. Both make it a low-cost alternative to mainstream global optimization methods, especially nature-inspired algorithms. This is particularly the case if the parameter space for the problem is set up in a reasonable way (e.g., not excessively large), and the likelihood that electrical characteristics of a randomly-generated design are not overly distorted is not excessively low.

A practical limitation (or, inconvenience) is that the feature-based approximations of the operating conditions need to be extracted from the EM simulated circuit responses, which is normally realized on case-to-case basis, although the respective implementations are transferrable within the same type of circuit responses (coupler, power divider, etc.). Automation of this process will be addressed elsewhere.

## Conclusion

In this paper, a simple and reliable procedure for computationally-efficient globalized design optimization of passive microwave circuit has been presented. The fundamental component of the algorithm is an inverse regression model constructed using information extracted from a pre-selected subset of randomly generated parameter vectors and the corresponding EM-simulated circuit characteristics. Inverse model rendition involves the response feature technology, which exploits weakly nonlinear relationships between the geometry and operating parameters of the system at hand. Numerical verification of the proposed procedure has been carried out using three microstrip components, including two miniaturized rat-race couplers, and a dual-band power divider. In each case, ten independent algorithm runs were executed to validate the efficacy of the method as well as repeatability of solutions. Satisfactory designs have been found in all executions, which was not the case for the benchmark algorithms, especially multiple-start local search, where inferior designs were produced for a considerable number of optimization runs. At the same time, the computational cost of our framework is significantly lower than that of state-of-the-art global optimizers (here, PSO). As a matter of fact, it is comparable to the cost of local gradient-based optimization. The methodology discussed in this work might be an attractive alternative to conventional global search methods, particularly nature-inspired algorithms, but also hybrid methods incorporating forward surrogate modelling methods. The major advantages include low computational complexity, global search capability, as well as the improved immunity to dimensionality and parameter range issues. Although demonstrated for microwave passives, the proposed algorithm might be generalized to other type of circuits, including amplifiers or mixers; however, this requires additional investigation. While extending the applicability of the presented approach, one should keep in mind that in the case of other microwave components, the major limitation factor might be that the assumption concerning weakly-nonlinear relation between the figures of interest and designable parameters may no longer hold. In the latter case, utilization of the inverse surrogates (at the global search stage) would not be as efficient as demonstrated for the passive circuits. This will be considered in the future work. On the other hand, the non-uniqueness issues may be detrimental to the algorithm performance as well.
